# PARot – assessing platelet-rich plasma plus arthroscopic subacromial decompression in the treatment of rotator cuff tendinopathy: study protocol for a randomized controlled trial

**DOI:** 10.1186/1745-6215-14-167

**Published:** 2013-06-11

**Authors:** Andrew Carr, Cushla Cooper, Richard Murphy, Bridget Watkins, Kim Wheway, Ines Rombach, David Beard

**Affiliations:** 1 NIHR Biomedical Research Unit, Nuffield Orthopaedic Centre, Oxford University Hospitals NHS Trust, Windmill Road, Headington, Oxford, OX3 7LD, UK

**Keywords:** Platelet-rich-plasma, Rotator cuff, Tendinopathies, Surgery, Growth factors

## Abstract

**Background:**

Platelet-rich plasma (PRP) is an autologous platelet concentrate. It is prepared by separating the platelet fraction of whole blood from patients and mixing it with an agent to activate the platelets. In a clinical setting, PRP may be reapplied to the patient to improve and hasten the healing of tissue. The therapeutic effect is based on the presence of growth factors stored in the platelets. Current evidence in orthopedics shows that PRP applications can be used to accelerate bone and soft tissue regeneration following tendon injuries and arthroplasty. Outcomes include decreased inflammation, reduced blood loss and post-treatment pain relief. Recent shoulder research indicates there is poor vascularization present in the area around tendinopathies and this possibly prevents full healing capacity post surgery (*Am J Sports Med***36**(6):1171–1178, 2008). Although it is becoming popular in other areas of orthopedics there is little evidence regarding the use of PRP for shoulder pathologies. The application of PRP may help to revascularize the area and consequently promote tendon healing. Such evidence highlights an opportunity to explore the efficacy of PRP use during arthroscopic shoulder surgery for rotator cuff pathologies.

**Methods/Design:**

PARot is a single center, blinded superiority-type randomized controlled trial assessing the clinical outcomes of PRP applications in patients who undergo shoulder surgery for rotator cuff disease. Patients will be randomized to one of the following treatment groups: arthroscopic subacromial decompression surgery or arthroscopic subacromial decompression surgery with application of PRP.

The study will run for 3 years and aims to randomize 40 patients. Recruitment will be for 24 months with final follow-up at 1 year post surgery. The third year will also involve collation and analysis of the data. This study will be funded through the NIHR Biomedical Research Unit at the Oxford University Hospitals NHS Trust.

**Trial registration:**

Current Controlled Trials: ISRCTN10464365

## Background

Platelet-rich plasma (PRP) is an autologous platelet concentrate. It is derived from patients’ own blood and mixed with an agent to activate platelet formation. PRP works by increasing the number of platelets, which release growth factors to help healing of a particular area [1]. Oral surgeons have used PRP for many years and it is now being widely used in orthopedic interventions. Studies have included comparisons of PRP injections with a steroid or analgesic injection or comparing PRP application during surgery with no PRP application.

Current clinical evidence suggests PRP could have beneficial effects on hard and soft tissue healing due to the presence of growth factors stored in the platelets [2]. The evidence also shows that PRP applications can be used to accelerate bone and soft tissue regeneration following tendon injuries and arthroplasty. Outcomes include decreased inflammation, reduced blood loss and post-treatment pain relief [3]. Previous studies assessing the use of PRP in orthopedics have involved plantar fasciitis, lateral epicondylitis, Achilles tendonitis and arthroplasty. However, there is little evidence regarding the use of PRP in shoulder pathologies.

Shoulder tendinopathies, such as rotator cuff impingement and tears, are an increasing problem in orthopedic practice and reportedly account for up to 70% of shoulder pain problems [4]. Impingement is the most frequent cause of shoulder pain in the general population. The term ‘rotator cuff tear’ refers to structural failure and tissue disruption in at least one of the four muscles and tendons that form the rotator cuff. Any tear that involves rotator cuff disruption that does not extend all the way through the tendon is termed a partial-thickness tear (PTT). PTTs are more prevalent than full-thickness tears [5]. Information is also lacking regarding the risk of progression of PTTs to full-thickness tears.

Impingement and rotator cuff tears are associated with progressive change in the shape of the acromion, with spurs forming at its anteroinferior margin. These spurs are thought to narrow the subacromial space, thereby making physical contact more likely, particularly in certain positions of the arm (for example, the painful arc) [4,6] and resulting in inflammation, pain and potentially some loss of function.

A high proportion of patients with impingement and partial-thickness tears will respond to conservative treatment [7]. The most common surgical intervention for impingement and partial-thickness tears is arthroscopic subacromial decompression (SAD). Indications for surgery include persistent and severe pain combined with functional restrictions that are resistant to conservative measures. Despite surgery being considered at this point, some reports show that surgery is no more effective than physiotherapy in the relief of pain when used in patients at this stage [8,9]. Recent studies indicate the poor vascularization present in the area around tendinopathies possibly prevents full healing capacity [10]. The application of PRP may help to revascularize the area and consequently promote tendon healing. Such evidence highlights an opportunity to explore the efficacy of PRP use in subacromial decompression surgery for rotator cuff pathologies.

The objective is to assess the effectiveness of treating subacromial impingement syndrome and partial-thickness rotator cuff tears with autologous platelet-rich plasma. We plan to compare the outcome of standard arthroscopic subacromial decompression surgery with the outcome of the same surgery plus the administration of autologous platelet-rich plasma.

## Methods/design

PARot is a parallel blinded superiority-type randomized controlled trial. The two groups are: arthroscopic subacromial decompression surgery and arthroscopic subacromial decompression surgery with application of PRP.

The study will run for 3 years. Recruitment will be for 24 months with final follow-up at 1 year post surgery. The final year will involve collation and analysis of the data. Only patients will be blinded as to whether they received PRP or not.

Patients identified for the study will have failed conservative treatment for their shoulder impingement or partial-thickness tear and will be listed for subacromial decompression surgery. Conservative treatment may include physiotherapy and injections of steroid and or local anesthetic. The routine step following failed conservative treatment would usually involve being put on the waiting list for surgery (SAD).

Patients will be identified in, and recruited from, the shoulder outpatient clinics at the Nuffield Orthopaedic Centre, Oxford University Hospitals (OUH) NHS Trust, Oxford. Diagnosis of shoulder impingement or partial-thickness tear will be confirmed with ultrasound in the clinic and the patient will be put on the waiting list for surgery. A surgical member of the research team will introduce the trial to the patient and refer them to a research nurse for recruitment.

Informed consent procedures, baseline assessments (including the Oxford shoulder score) and randomization are performed up to 3 days prior to surgery due to organizational requirements in relation to operating schedules and required equipment. Randomization is undertaken using sealed envelopes based on a simple computer-generated randomization list.

Routine perioperative care will proceed and the patient will have their operation as planned.

**Figure 1:**
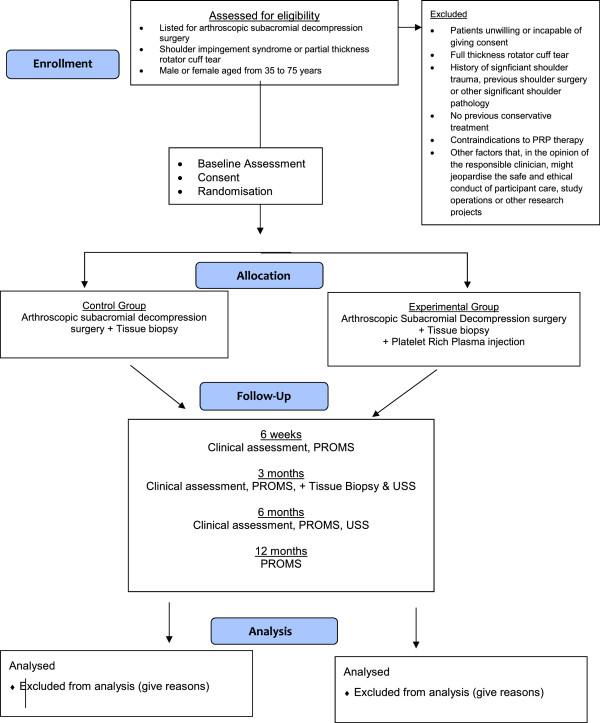
**Study flow chart.** PROMS, patient-reported outcome measures.

As randomization occurs very close to the point of treatment, patients will be followed up in clinic at 6 weeks and at 3 and 6 months post treatment. Patients will be blinded to the treatment they are allocated to. Assessors and surgeons are unable to be blinded in this instance. All patients will remain on the waiting list for surgery as per standard care and may have their surgery at the planned date. A summary of the study design is portrayed in the flow chart (Figure 1).

PARot study assessments will include patient-reported outcome measures (PROMs), histological examinations, functional assessments and patient satisfaction and health economic questions. Evaluation of routine basic blood tests will also be included in the PARot assessments. A full blood count (taken as part of routine preoperative assessment and not a study intervention) will indicate the presence of clotting substances and any PRP contraindications the patient may have.

Patients will be required to attend a clinic appointment for assessments at 6 weeks and at 3 and 6 months post treatment. A postal questionnaire (containing the Oxford shoulder score, Oxford satisfaction index and EQ 5D) will also be sent out at 12 months post treatment. Clinical assessments and any adverse reactions or side effects can be documented. Table 1 outlines the outcome measures and their correlating time points.

**Table 1:** Outcome assessments

	**Baseline**	**6 weeks**	**3 months**	**6 months**	**12 months**
**Oxford shoulder score**	X	X	X	X	X
**Tissue biopsy**	X^a^		X^b^		
**Pain diary**		X			
**Clinical examination**	X	X	X	X	
**Oxford satisfaction index**	X	X	X	X	X
**EQ 5D**	X	X	X	X	X
**Complications**	X	X	X	X	X
**Venous blood sampling**	X				
**Ultrasound**	X		X	X	

^a^ Ultrasound-guided biopsy during surgery.

^b^ Ultrasound-guided biopsy at follow-up visit.

The primary outcome measures are the Oxford shoulder score (OSS) assessed at 12 months and the histological assessments of the biopsy tissue. The Oxford shoulder score is a condition-specific questionnaire providing a total score based on the patient’s subjective assessment of pain and activities of daily living (ADL) impairment. The range of available scores is 0 (worst) to 48 (best) [11]. The score is reliable and valid. The questionnaire will be administered to patients at the baseline and during their follow-up visits.

Secondary outcomes involve basic histological tests to assess vascularity, antibodies and neural tissue and cell creation [12]. Tissue samples will be taken at the baseline (during surgery) and again 3 months later. Secondary outcomes also include radiological and clinical assessment of impingement syndrome to assess the progression of any cuff tear. Data related to patient satisfaction and resource use involved in each treatment arm will also be collected at the baseline and again at follow-up visits.

The PARot study will include patients diagnosed with subacromial impingement syndrome or a partial-thickness tear who have failed conservative treatment. The inclusion criteria are the following:

• Patients with shoulder impingement syndrome or a partial-thickness rotator cuff tear.

• Diagnosis confirmed using ultrasound scan by a trained member of the research team.

• Failed conservative treatment.

• Listed for arthroscopic subacromial decompression surgery.

• Male or female, aged 35 to 75 years old.

Patients may not enter the study if any of the following apply:

• Full thickness rotator cuff tears.

• Participants with a history of significant trauma. (fracture, dislocation, instability and so on), surgery, osteoarthritis or other significant pathology of the affected shoulder not related to the rotator cuff.

• Patient is unable to consent.

• No conservative treatment.

• Previous surgery on affected surgery.

• Contraindications to PRP (listed below).

PRP contraindications:

• History of diabetes mellitus.

• Platelet abnormality or platelet count <100 × 10^9^/l.

• Hematological disorder.

• Serum hemoglobin <11 g/dl.

• Use of systemic cortisone.

• Use of any anticoagulant.

• Evidence of gangrene, ulcers or peripheral vascular disease.

• History of hepatic or renal impairment or dialysis.

• Patient is known to have a psychological, developmental, physical, emotional or social disorder that may interfere with compliance with study requirements.

• History of alcohol or drug abuse.

• Patient has a religious or cultural conflict with the use of platelet gel or blood products.

• Patient has inadequate venous access for blood draw.

• Patient is currently receiving or has received radiation or chemotherapy within the last 3 months prior to the study.

• Female participants who are pregnant, lactating or planning pregnancy during the course of the study.

• Any other significant disease or disorder that, in the opinion of the investigator, may either put the participant at risk because of participation in the study, or may influence the result of the study, or the participant’s ability to participate in the study.

## Discussion

### Definition of end of study

The end of the trial is the date of the last visit or telephone follow-up of the last participant.

### Discontinuation or withdrawal of participants from study treatment

Each patient has the right to withdraw from the study at any time. In addition, the investigator may discontinue a patient from the main analysis at any time if the investigator considers it necessary for any reason including:

• Development of any PRP contraindications.

• Significant protocol deviation.

• Significant non-compliance with treatment regimen or study requirements.

• An adverse event that results in an inability to continue to comply with study procedures.

• Disease progression that requires discontinuation of the study medication or results in inability to continue to comply with study procedures.

• Lost to follow-up.

No procedures or observation will continue to be required following withdrawal from the trial. Data collected before withdrawal from the trial will be used in the statistical intention-to-treat analyses and analysis of all participants receiving the study treatment.

### Subacromial decompression surgery

Subacromial decompression surgery is performed under general anesthetic. The procedure involves insertion of an arthroscope into the glenohumeral joint where the joint surface is inspected along with the intra-articular portion of the long head of the biceps and the joint surface of the rotator cuff tendons. Once this has been performed the arthroscope is removed and inserted into the subacromial bursa, which lies outside the rotator cuff tendons and beneath the acromion process of the scapula. In the bursa, the acromion and superior surface of the rotator cuff are assessed to ensure the coracoacromial ligament and the Acromioclavicular (AC) joint remains intact. The projecting under surface of the distal part of the acromion is resected. The intervention is considered to be a well-established and well-documented procedure. Patients with full cuff tears noted at the time of surgery will not be eligible and will be excluded from the trial at operation.

### Subacromial decompression surgery with autologous platelet-rich plasma

This group will receive the same operation as described above plus an autologous platelet-rich plasma concentrate injection into the rotator cuff tendon during surgery. PRP can be applied by a variety of methods: as a gel, as an injection, as a sutured film during surgery, sprayed directly onto repaired tissue or injected subcutaneously. This study proposes the use of a gel sprayed directly onto the decompression area.

Specific preparation techniques will be stipulated by the company providing the PRP device. Described below is a guideline for the preparation involved in administering PRP, which was taken from the protocol of the PATH study (REC Reference: 09/H0605/78), also run in this department.

PRP is prepared using a sterile technique in the theatre. First, 50 ml of venous blood will be withdrawn from the patient into a syringe. The syringe will be attached to the selective platelet separator device and the blood is transferred into the device. The device will separate platelets and remove red blood cells and white blood cells. The result is 5 ml to 6 ml of platelet-rich concentrate (PRC), which will be collected in an attached syringe [13].

The 5-ml syringe containing the concentrate is attached via a dual injection kit to another syringe containing bovine thrombin (200 units) and 1 ml of 10% calcium chloride (activator). The dual syringes are used to inject the PRC into the subacromial space.

Reviews in oral surgery recommend that the derivation of PRP must be a sterile process. Platelets must not be lysed or damaged in any way, as damaged platelets may not release their growth factors. Studies that suggest there is no benefit to PRP application may have suffered from poor preparation of the PRP solution [14]. It is therefore important to send excess PRP to the laboratory for platelet analyses and quality assessment [15]. Data obtained from tendon tissue samples from all patients will also provide valuable information regarding the integrity of the tendon including any effects PRP may have on this.

### Tendon biopsies

Tendon biopsies for histological assessments will be taken during surgery for both groups of patients. This will involve a small amount of tendon tissue (the size of a small grain of rice) excised using a 14G core biopsy needle under ultrasound guidance. This will be taken in a sterile manner as part of the surgical procedure before the application of the PRP.

A further biopsy will be taken from both groups of patients during their 3-month follow-up visit. This biopsy will be taken using an ultrasound guidance technique and a biopsy needle in exactly the same manner as performed at surgery, under sterile conditions. Local anesthetic will be infiltrated around the area of the biopsy to numb the region before the tissue sample is taken. This technique has been successfully trialed and used in previous studies conducted by this research group (ethics ref: 09/H0605/111 and 09/0605/78).

### Venous blood sampling

A sample of 18 ml of venous blood will be obtained from the patient for analysis and inclusion in the Oxford Musculoskeletal BioBank project (ethics ref: 09/H606/11). The sample will be taken prior to commencement of surgery once the patient is anesthetized. For those patients in the PRP group this sample will be taken using the same needle as for the blood taken for making up the PRP solution. In this way patients will be blinded to their group allocation as both groups will have a single venepuncture site and the sampling volume will be unknown to the patient as they will have been anesthetized prior to venepuncture.

### Safety

Adverse events have not been reported well in previous studies involving PRP. This study will record expected adverse events and will report unexpected events that are related to the patients’ participation in the study. PARot’s final report will include a section on adverse events even if this is just to confirm that none were reported.

### Statistics and analysis

The planned study examines a continuous response variable (OSS) from independent control and experimental subjects. In previous studies the response within each subject group was found to be normally distributed with a standard deviation of 3 points. If the true difference (or the meaningful clinical difference) in the experimental and control means were 3 points on the OSS, we would need to study 17 experimental subjects and 17 control subjects to be able to reject the null hypothesis that the population means of the experimental and control groups are equal with probability (power) 0.8. The Type I error probability associated with this test of this null hypothesis is 0.05 (α). To allow for losses to follow-up we aim to recruit 20 patients in each arm.

### Analysis of endpoints

Reporting of outcomes will follow CONSORT Guidelines. Full details of the planned analysis will be detailed in the statistical analysis plan and approved by the trial team prior to any analysis being undertaken.

### The Oxford shoulder score

The OSS at 12 months is the primary outcome measure and the primary hypothesis is based upon this evaluation. The design is a simple two-group cross-sectional comparison. The data will be first checked for normality using histograms and Shapiro–Wilk tests. Independent *t*-tests (assuming normal distribution and equal variance) will then be performed to test the hypothesis that there is no difference in the mean OSS between the two groups. The null hypothesis will be rejected for significance set at α = 0.05.

### Histology assessments

The tendon structure will be examined with basic histology by staining specimens with hematoxylin and eosin, toluidine blue and Congo red. Immunohistochemical analysis will also be performed using specific antibody markers such as vascular endothelial markers (CD 34), macrophage-associated markers (CD 68) [12] as well as neural tissue (S100) and cell proliferation (PCNA). Statistical analysis with *t*-tests will be used with significance set at α = 0.05.

### Secondary outcome measures

Descriptive statistics will be presented for the secondary endpoints but no formal significance tests will be performed. The sample size is insufficient to test hypotheses for this categorical data but some valuable indication of effect may be observed.

### Ethics

The study has obtained approval from the National Research Ethics Service, Oxfordshire, REC C in October 2010 (0/H0G0OIO0).

## Trial status

The trial is currently recruiting patients and has completed follow-up on 40 patients.

## Abbreviations

ADL: Activities of daily living; NHS: National Health Service; NIHR: National Institute of Health Research; OSIRIS: Orthopaedic Surgical and Interventional Research Group; OSS: Oxford shoulder score; OUH: Oxford University Hospitals; PROMS: Patient-reported outcome measures; PRC: Platelet-rich concentrate; PRP: Platelet-rich plasma; PTT: Partial-thickness tear; SAD: Subacromial decompression; AC joint: Acromioclavicular joint; USS: Ultrasound scan; BRU: Biomedical Research Unit.

## Competing interests

The authors declare that they have no competing interests.

## Authors’ contributions

All of the authors contributed to the design and development of the trial protocol. AC, DB and CC were responsible for writing this manuscript. IR will oversee the statistical analysis. All authors read and approved the final manuscript.

## Authors’ information

AC is the Nuffield Professor of Orthopaedic Surgery and the director of the National Institute for Health Research Musculoskeletal Biomedical Research Unit (NIHR-BRU) in Oxford. DB is a director of the Orthopaedic Surgical and Interventional Research Group (OSIRIS) within the Nuffield Department of Orthopaedics, Rheumatology and Musculoskeletal Sciences at the University of Oxford. RM is completing his DPhil, CC is the OSIRIS trials manager, and KW and BW are specialist shoulder research nurses at the OUH NHS Trust.
